# Development of three-dimensional primary human myospheres as culture model of skeletal muscle cells for metabolic studies

**DOI:** 10.3389/fbioe.2023.1130693

**Published:** 2023-03-23

**Authors:** Andrea Dalmao-Fernandez, Aleksandra Aizenshtadt, Hege G. Bakke, Stefan Krauss, Arild C. Rustan, G. Hege Thoresen, Eili Tranheim Kase

**Affiliations:** ^1^ Section for Pharmacology and Pharmaceutical Biosciences, Department of Pharmacy, University of Oslo, Oslo, Norway; ^2^ Hybrid Technology Hub Centre of Excellence, Faculty of Medicine, University of Oslo, Oslo, Norway; ^3^ Department of Pharmacology, Institute of Clinical Medicine, University of Oslo, Oslo, Norway

**Keywords:** skeletal muscle, myosphere, energy metabolism, metabolic disorders, 3D cell model, muscle spheroid

## Abstract

**Introduction:** Skeletal muscle is a major contributor to whole-body energy homeostasis and the utilization of fatty acids and glucose. At present, 2D cell models have been the most used cellular models to study skeletal muscle energy metabolism. However, the transferability of the results to *in vivo* might be limited. This project aimed to develop and characterize a skeletal muscle 3D cell model (myospheres) as an easy and low-cost tool to study molecular mechanisms of energy metabolism.

**Methods and results:** We demonstrated that human primary myoblasts form myospheres without external matrix support and carry structural and molecular characteristics of mature skeletal muscle after 10 days of differentiation. We found significant metabolic differences between the 2D myotubes model and myospheres. In particular, myospheres showed increased lipid oxidative metabolism than the 2D myotubes model, which oxidized relatively more glucose and accumulated more oleic acid.

**Discussion and conclusion:** These analyses demonstrate model differences that can have an impact and should be taken into consideration for studying energy metabolism and metabolic disorders in skeletal muscle.

## 1 Introduction

Skeletal muscle comprises approximately 40% of the body’s total mass, thereby being a marked contributor to whole-body energy homeostasis including the utilization of lipids and glucose (Frontera and Ochala, 2015). Furthermore, the skeletal muscle is one of the most important insulin-sensitive organs in the body, accounting for more than 80% of insulin-stimulated glucose disposal (DeFronzo, 2004). In the fed state, the increased availability of plasma glucose stimulates glucose oxidation and fatty acid synthesis, whereas fatty acid oxidation increases during fasting or sustained exercise when energy demand is higher (Kelley et al., 1990; Henriksson, 1995). Ability to readily switch from fatty acids to glucose as the primary source of fuel during times of caloric abundance or deficit has been described as metabolic flexibility and is a characteristic of healthy skeletal muscle (Goodpaster and Sparks, 2017). However, development of metabolic disorders reduces the efficiency of switching between glucose and fatty acid metabolism. Cellular impairments caused by this loss of flexibility are also associated with reduced lipid oxidation promoting accumulation of lipids in skeletal muscle, which can interfere with insulin signaling and function (Galgani et al., 2008; Goodpaster and Sparks, 2017). This significantly impacts energy metabolism and could be a key to understanding the alterations attributed to skeletal muscle in obesity and type 2 diabetes.

At present, the use of 2D cell models is widespread as a model system for high-throughput drug screening since compounds added to cells can easily access the target with a homogenous distribution (Gholobova et al., 2018). Conventional 2D cell cultures are well known and have been widely employed for describing cellular processes (growth, proliferation, and differentiation), and studying energy metabolism and metabolic disorders during the last years (Shen et al., 2021). However, since several parameters measured in 2D muscle cell models are not comparable to those found *in vivo*, such as mRNA expression, glucose/fatty acid uptake and oxidation measurements, cellular insulin response, fiber types, cell senescence, cell differentiation, drug assimilation, resistance and cytotoxicity, transferability of results generated is limited (Aas et al., 2013; Ferrari et al., 2022). 2D cell models thus represent a system where altered or absent cell communications do not necessarily reproduce the microenvironment and complexity of biological systems in the body (Shen et al., 2021). As a result, global changes in phenotype, metabolism and functionality of cells in existing 2D cultures provide very limited/low predicting power for the *in vivo* drug efficacy, toxicity and development of related disorders (Hess et al., 2010; Antoni et al., 2015). To complement these results in 2D, the use of three-dimensional (3D) cell culture models such as tissue engineering, organ-on-a-chip technologies or basic cell constructs (organoids/spheroids) could replicate more accurately *in vivo* conditions, covering the gap between *in vitro* cell culture, animal models and human clinical trials providing more physiologically relevant data (Weinberger et al., 2017). Thus, the creation of 3D tissues from human cells may increase the physiological relevance of *in vitro* cell models and could possibly reduce live animal experiments in the fields of pathophysiology, pharmacokinetics, toxicity and drug delivery studies (Fitzgerald et al., 2015; Zuppinger, 2019; Shen et al., 2021).

Accordingly, new methods have been developed to assemble 3D cell cultures. Several of these techniques can be divided into scaffold-based 3D cell cultures (often referred to as organoids) or scaffold-free tissue-engineered materials (spheroids, spheres or aggregates) (Zuppinger, 2019; Jiang et al., 2022). For the first type of 3D culture, cells are usually anchored or placed in biomaterials or synthetic polymers, simulating an extracellular matrix (ECM), however, application of scaffolds limits and increases cost of analysis in such models (Costa et al., 2016; Zuppinger, 2019; Shen et al., 2021). Cellular spheroids (scaffold-free systems) represent easier to scale up and more uniform 3D cell culture models. In the past years, novel strategies such as hanging drop, ultra-low attachment plate, spinner culture and microfluidics have been developed to enable the generation of spheroids *via* aggregation of cells in microcavities (microwells). These systems would provide a natural physical obstacle that facilitates cell-to-cell interaction (Antoni et al., 2015; Ryu et al., 2019; Zuppinger, 2019; Gunti et al., 2021; Kang et al., 2021).

In the field of muscle research, muscle bioengineering has significantly improved the generation of 3D constructs *in vitro* over the last few years (Maffioletti et al., 2018). They are mainly originated as scaffold-based systems from immortalized cells or primary muscle cells from other species (murine, porcine) (Westerman et al., 2010; Westerman, 2015; Aguanno et al., 2019), human embryonic stem cells, or human induced pluripotent stem cells (Bremner et al., 2022; Raffa et al., 2022), and are mainly used in drug discovery or muscle function related diseases (exercise, force and muscle strength (contractility) fields) (Bremner et al., 2022; Jiang et al., 2022). In contrast to scaffold-based systems with a high cost and difficulty to upscale, the creation of muscle spheroids (myospheres) provides a simple and reproducible approach to achieve 3D structures. Several examples have been described in the literature creating myospheres from immortalized muscle cells, hESC or hiPSC lines (Maffioletti et al., 2018). However, a limited number of experiments with myospheres models using human primary skeletal muscle cells established from human biopsies have been published. In these cases, myospheres originated from human primary skeletal muscle cells have only been used to evaluate the myogenic properties of the cells. As a result, satellite muscle cells have been expanded in suspension as myospheres and then, dissociated and cultured as myosphere-derived cells in 2D (Westerman et al., 2010; Wei et al., 2011; Westerman, 2015; Aguanno et al., 2019; Naldaiz-Gastesi et al., 2019). The reasons behind this could be low access to human muscle biopsies or advantages of working with immortalized cells (longer lifespan, easy handling) (Maqsood et al., 2013). However, a recently published comparison between muscle primary and immortalized cell models has revealed deeply different transcriptomic profiles and metabolic behaviors among models which must be considered for appropriate use depending on scientific hypotheses and biological relevance (Abdelmoez et al., 2020).

Therefore, the aim of this study was to develop a simple, self-aggregating, low-cost, scaffold-free, and reproducible method to establish a 3D skeletal muscle cell model (myospheres) from biopsy-derived primary human satellite cells to study molecular mechanisms and alterations in skeletal muscle that are characteristic of metabolic disorders.

## 2 Material and methods

### 2.1 Material

Corning^®^CellBIND^®^ tissue culture plates (96-well and 6-well) were purchased from Corning (Schiphol-Rijk, the Netherlands). Dulbecco’s Modified Eagle’s Medium (DMEM)-Glutamax™ low glucose, Dulbecco’s Phosphate Buffered Saline (DPBS; without Ca^2+^ and Mg^2+^), penicillin-streptomycin (10,000 IE/mL), amphotericin B, human epidermal growth factor (hEGF), trypsin-EDTA, fetal bovine serum (FBS), Nunclon™ Sphera™ 96 Well Round (U) Bottom Plate Low-Attachment Surface, Paraformaldehyde (PFA), LIVE/DEAD™ Viability/Cytotoxicity Kit, Alexa Fluor™ 488 Phalloidin and Image-iT™ Green Hypoxia Reagent were bought from Thermo Fisher Scientific (Waltham, MA, US). TRIzol™ Reagent, Power SYBR^®^ Green PCR Master Mix, High-Capacity cDNA Reverse Transcription Kit, MicroAmp^®^ Optical 96-well Reaction Plate, MicroAmp^®^ Optical Adhesive Film, primers for PCR, and Nunc™ Cell and Culture Treated Flasks with Filter Caps were also bought from Thermo Fisher Scientific. Insulin (Actrapid^®^Penfill^®^ 100IE/mL) was from NovoNordisk (Bagsvaerd, Denmark). D-[^14^C(U)]glucose (3.0 mCi/mmol) and [1-^14^C]oleic acid (OA, 59.0 mCi/mmol) were from PerkinElmer NEN^®^ (Boston, MA, US). Ultima Gold™ XR, UniFilter^®^-96 GF/B microplates, 96-well Isoplate^®^, and TopSeal^®^-A transparent film were obtained from PerkinElmer (Shelton, CT, US). 4-(2-hydroxyethyl)-1-piperazineethanesulfonic acid (HEPES), bovine serum albumin (BSA), dexamethasone, gentamicin, L-glutamine, L-carnitine, trypan blue 0.4% solution, D-glucose, oleic acid (OA, 18:1, n-9), chloroform, Isopropanol, Triton-X100 and Hoechst 33,258 were purchased from Sigma-Aldrich (St. Louis, MO, US). PromoCell medium and PromoCell Supplement Mix were obtained from Promocell (Heidelberg, Germany). CellTiter-Glo 3D^®^ cell viability assay was from Promega (Madison, WI, US). Secondary antibodies: Alexa Fluor^®^ 647 AffiniPure Donkey Anti-Mouse IgG (H + L) and Cy™3 AffiniPure Donkey Anti-Rabbit IgG (H + L) were acquired from Jackson ImmunoResearch (West Grove, PA, US). Primary antibodies: rabbit monoclonal Ab to collagen type I and mouse monoclonal antibody to vimentin were bought from Abcam (Cambridge, United Kingdom). Sodium dodecyl sulfate (SDS) and Bio-Rad protein assay were purchased from Bio-Rad (Hercules, CA, US).

### 2.2 Methods

#### 2.2.1 Human skeletal muscle cells: Cell isolation and culture

Muscle biopsies were obtained from *musculus vastus lateralis* of eight healthy males and 4 healthy females at either Faculty of Social and Health Sciences, Section for Health and Exercise Physiology, Inland Norway University of Applied Sciences or at the Norwegian School of Sport Sciences, Oslo. The donors were 21–57 years old and the body mass index average was 29.2 ± 7.4 kg/m^2^ (mean ± SD). All donors were informed and signed a written consent before the biopsy. The studies involving human participants have been approved by the Regional Committees for Medical and Health Research Ethics in Norway (REK South-East) (reference numbers 2011/2,207 and 11,959). All the procedures were conducted according to principles expressed in the Declaration of Helsinki.

The human skeletal muscle cells (satellite primary cells) were isolated as previously described (Lund et al., 2018), based on the modified method of Henry et al. (1995) (Gaster et al., 2001a; Gaster et al., 2001b). Isolated satellite primary cells were escalated in PromoCell medium supplemented with PromoCell Supplement Mix, penicillin (25 IU), streptomycin (25 μg/mL) and amphotericin B (1.25 μg/mL) and stored as myoblasts in liquid nitrogen. Before the experiments, myoblasts were proliferated in Nunc™ flasks up to 80%–90% confluence in proliferation medium (DMEM-Glutamax™ (5.5 mmol/L glucose) supplemented with 10% FBS, HEPES (25 mmol/L), gentamicin (50 ng/mL), penicillin (25 IU), streptomycin (25 μg/mL), amphotericin B (1.25 μg/mL), hEGF (10 ng/mL), dexamethasone (0.39 μg/mL), and 0.05% BSA) and maintained at 37°C in a humidified 5% CO_2_ incubator. During the proliferation phase, media was changed every second day.

For the 2D cell-model experiments, 7 × 10^3^ myoblasts per well were cultured in a 96-well Corning^®^CellBIND^®^ tissue culture plate, grown to 70%–80% confluence and differentiated to multinucleated myotubes by replacing proliferation medium with differentiation medium [DMEM-Glutamax™ (5.5 mmol/L glucose) containing 2% FBS, 25 p.m. insulin, HEPES (25 mmol/L), amphotericin B (1.25 μg/mL), gentamicin (50 ng/mL), penicillin (25 IU), and streptomycin (25 μg/mL)]. The differentiation phase was carried out for at least 10 days unless stated otherwise.

#### 2.2.2 3D myosphere model formation: Differentiation of myospheres

The establishment of the 3D myosphere model was performed using a Nunclon™ Sphera™ 96-well plate whose main characteristics are ultra-low attachment treatment (ULA) and a U-shaped bottom. Between 2.5 and 3.5 × 10^4^ myoblasts per well were seeded in a Nunclon™ Sphera™ plate in proliferation medium. Thereafter, the plates were placed in the incubator and the cells in suspension aggregated by natural sedimentation in the middle/bottom of the plate and formed spheres within the next hours (around 4–8 h). 24 h after the formation of the spheres, i.e., the day after sedimentation, proliferation media was changed to differentiation media (day 0), and the differentiation phase was carried out for at least 10 days. To analyze and compare both cell models, composition of the media was the same between 2D and 3D cell model cultures (Supplementary Figure S1).

#### 2.2.3 Phase-contrast morphological analysis

The morphology of the obtained myospheres was monitored for several days by the open software AnaSP (Piccinini, 2015) after acquisition of phase-contrast photos by Olympus CKX41 inverted microscope and Olympus SC30 camera (Olympus Life Science, Japan). In brief, software converted images (one photo-one spheroid) into grayscale and segmented the myospheres to analyze a binary mask where morphological parameters are extracted. Around a total number of 90 images were analyzed twice during different time points (days 3, 7, 10, 12, 14, 17, and 21) with at least 3-4 different spheroid pictures in differentiation phase.

#### 2.2.4 Myospheres viability

The viability of the myospheres was performed by two different approaches: relative ATP content (luminescence) and LIVE/DEAD cell staining.

##### 2.2.4.1 CellTiter-Glo 3D^®^ cell viability assay

Myospheres were cultured as explained above. Relative ATP content was analyzed on days 0, 3, 7, and 10 of the differentiation phase and at least 12 single spheroids were analyzed separately at each time point. On each day of measurement, 50 µL of media with a single spheroid were transferred to a well of a 96-well Isoplate^®^ sealed with a white adhesive bottom seal for a 96-well. Thereafter, an equal volume of room-tempered CellTiter-Glo^®^ 3D Reagent was added to each well, mixed vigorously for 5–10 min, incubated in darkness for 20–25 min, and ATP content (RLU) was measured using a 2,450 MicroBeta^2^ liquid scintillation counter (PerkinElmer) at 1 measurement per second. ATP content was then normalized to day 0 and represented as a percentage (%).

##### 2.2.4.2 LIVE/DEAD cell staining

Myospheres were stained with LIVE/DEAD^®^ Viability/Cytotoxicity Assay Kit to assess the amount of live and dead cells. The probe calcein acetoxymethyl (AM) was used to signal intracellular esterase activity of live cells. The probe ethidium homodimer-1 (EthD-1) was used as a marker of cell death since it can only cross damaged membranes of compromised viable cells and binds to nucleic acids producing fluorescence. Staining was performed on days 0 and 10 of the differentiation phase, analyzing a total of 12 spheroids per day. Briefly, 12 spheroids were pooled and sunk in the bottom of an Eppendorf microcentrifuge tube. Thereafter, media was changed to 1 mL of PBS with calcein AM and EthD-1 ratio 1:1,000 and incubated for 30 min. Previous to the image acquisition, PBS with the probes was changed for PBS and spheroids were transferred to a 6 MW plate. Between 5 and 8 images were taken by Zeiss Axio Vert. A1 microscope equipped with Axiocam 202 mono camera and Colibri 7 Illumination module and analyzed by open-source software FIJI (ImageJ) (Schindelin et al., 2012).

#### 2.2.5 Hypoxia core

The oxygen gradient in the core of the myospheres was studied by Image-iT™ Green Hypoxia Reagent. 12 independent live myospheres at 0 and 10 days of differentiation were transferred to a 6-well Corning^®^ tissue culture plate and stained for 1 h at 37°C with Image-iT™ Green Hypoxia Reagent at a final concentration of 5 µM in cell medium. Subsequently, medium was replaced with a fresh cell medium, and images were taken after 24 h. Before image acquisition, nuclear counterstaining was performed with 1 μg/mL Hoechst 33,258. Confocal microscopy was performed as detailed previously and images were analyzed using FIJI (ImageJ) (Schindelin et al., 2012).

#### 2.2.6 Immunofluorescence staining and microscopy

The internal organization of muscle cells into 3D sphere shape was monitored during the differentiation phase (0, 3, 6, and 10 days) by immunofluorescence staining. A total number of 8 spheroids per donor and time-point were fixed in 4% (w/v) PFA for 30 min at room temperature on an orbital shaker. Permeabilization and blocking were performed by incubation in PBS with 1% (m/v) BSA, 0.5% (v/v) Triton-X100% and 0.02% (m/v) SDS overnight at 4°C, on an orbital shaker. Staining with primary antibodies was performed for 30 h (at 4°C) with subsequently 24 h incubation with secondary antibodies diluted in blocking solution. The utilized antibodies and molecular probes are listed in Table 1. For tissue clearance organoids were incubated overnight in 88% (v/v) glycerol in PBS. Confocal microscopy was performed on a Zeiss 700 laser scanning confocal microscope using standard filter sets and laser lines with a ×20 air, and ×40 oil immersion objective. Images were acquired using Zen software (Zeiss) as Z-stacks with 5 µm spacing between stacks when using ×20 objective, and 2 μm–×40 oil objective. Confocal images were analyzed using the open-source software FIJI (ImageJ) (Schindelin et al., 2012) and are displayed as a resulting Z-stack of half of the myosphere.

**TABLE 1:** List of reagents for immunostaining.

	Name	Dilution	Catalog number
Primary antibodies	Rabbit monoclonal antibody to collagen type I	1:300	138,492
	Mouse monoclonal antibody to vimentin	1:300	ab8978
Secondary antibodies	Cy™3 AffiniPure Donkey Anti-Rabbit IgG (H + L)	1:400	711-165-152
	Alexa Fluor^®^ 647 AffiniPure Donkey Anti-Mouse IgG (H + L)	1:400	715-605-150
F-actin	Alexa Fluor™ 488 Phalloidin	1:500	A12379
Nuclear counterstaining	Hoechst 33,258	1 μg/mL	

#### 2.2.7 RNA isolation and quantitative real-time PCR

Gene expression of differentiation markers was analyzed by quantitative real-time PCR. 2D and 3D cell models were cultured as detailed above. A total of 60 wells per differentiation time point (0 and 10 days) were harvested by using TRIzol. Shortly, 60 well of muscle 2D cells were washed with PBS, detached by 3 min incubation with 20 µL of trypsin, and pooled in a 1.5 mL Eppendorf microcentrifuge tube. A cell pellet was formed and washed with PBS by 1,500 rpm centrifugation and 1 mL of TRIzol was added for RNA isolation. In parallel, 60 single spheroids were pooled, washed with PBS, and sunk by centrifugation in a 1.5 mL Eppendorf microcentrifuge tube. 1 mL of TRIzol was added and the sample was homogenized by IKA^®^ Ultra-Turrax^®^ tissue homogenizer (Sigma-Aldrich, St. Louis, MO, US) for the complete RNA isolation. According to manufacturer´s protocol, 0.2 mL of chloroform added directly to 1 mL of TRIzol was used for lysis and separation of the aqueous phase containing RNA. 0.5 mL of isopropanol was mixed to precipitate RNA. Consequently, the RNA pellet was washed with 75% ethanol and resuspended in 15 µL of RNase-free water.

0.3 µg of RNA was reversely transcribed into cDNA by High-Capacity cDNA Reverse Transcription using a PerkinElmer Thermal Cycler 9,600 following the manufacturer´s instructions. RT-PCR was performed in StepOnePlus™ Real-Time PCR system (Thermo Fisher Scientific) using Power SYBR^®^ Green PCR Master Mix. Sequence primers are listed in Table 2, and probe and PCR conditions are available upon request. The gene expression was normalized to the housekeeping control gene ribosomal protein lateral stalk subunit P0 (*RPLP0*).

**TABLE 2:** Primer’s sequence.

	Forward	Reverse
*RPLP0*	TGG​CCT​CAT​AGA​CAC​AGA​AAC​AG	CAG​GGC​ACA​TTC​CTC​CTT​TG
*MYOG*	GGA​CTG​GAC​GCC​CTC​ATT​C	CGCTCTGGTCCCCTGCTT
*MYOD*	GCG​CCC​AAA​AGA​TTG​AAC​TTA	CCG​CCT​CTC​CTA​CCT​CAA​GA
*MYH2*	AAG​GTC​GGC​AAT​GAG​TAT​GTC​A	CAA​CCA​TCC​ACA​GGA​ACA​TCT​TC
*MYH7*	CTC​TGC​ACA​GGG​AAA​ATC​TGA​A	CCC​CTG​GAC​TTT​GTC​TCA​TT
*SLC2A1*	CAG​CAG​CCC​TAA​GGA​TCT​CTC​A	CCGGCTCGGCTGACATC
*SLC2A4*	ACC​CTG​GTC​CTT​GCT​GTG​TT	ACC​CCA​ATG​TTG​TAC​CCA​AAC​T
*CD36*	AGT​CAC​TGC​GAC​ATG​ATT​AAT​GGT	CTG​CAA​TAC​CTG​GCT​TTT​CTC​AA

*RPLP0*, ribosomal protein lateral stalk subunit P0; *MYOG*, myogenin; *MYOD*, myogenic differentiation 1; *MYH2*, myosin heavy chain 2; *MYH7*, myosin heavy chain 7; *SLC2A1*, solute carrier family 2 member 1; *SLC2A4*, solute carrier family 2 member 4; *CD36*, CD36 molecule.

#### 2.2.8 Functional experiment: Substrate oxidation assay

A substrate oxidation assay was performed to evaluate metabolic activity of the myospheres. Glucose and oleic acid (OA) metabolisms were studied using labeled substrates, D-[^14^C (U)] glucose and [1-^14^C]OA, respectively. On day 10 of differentiation, both types of cell models, 2D and 3D, from three independent donors were incubated with these substrates for 4 h following the previously described protocol from Wensaas et al. (Wensaas et al., 2007). In short, differentiation media was changed for 50 µL of either glucose radioactive substrate [0.5 μCi/mL, 200 μM prepared in DPBS with BSA (10 μM) and HEPES (10 mmol/L)] or OA (0.5 μCi/mL, 100 µM prepared in DPBS with HEPES (10 mmol/L), L-carnitine (1 mmol/L) and BSA (40 μM). Subsequently, 96 well plates were mounted with a 96-well UniFilter^®^ microplate, previously activated by the addition of 1 M NaOH in the trapping device. After 4 h incubation, cells and myospheres were washed with PBS and harvested in 0.1 M NaOH. ^14^CO_2_ trapped in the filter, produced from the metabolized substrate, and cell-associated (CA) radioactivity from glucose or OA, were measured by addition of scintillation fluid and counted on a 2,450 MicroBeta^2^ scintillation counter. The obtained data were normalized by the protein content measured by Bio-Rad protein assay using a VICTOR™ X4 Multilabel Plate Reader (PerkinElmer).

The total uptake of each substrate was calculated as the sum of both ^14^CO_2_ and CA radioactivity (CO_2_+CA), while fractional substrate oxidation was calculated as the proportion of uptake substrate that goes to oxidation (CO_2_/CO_2_+CA).

#### 2.2.9 Statistical analysis

Data are presented as mean ± standard error of the mean (SEM) from three different donors with a minimum of three observations per donor unless stated otherwise. Statistical analyses were performed using GraphPad Prism software version 9 (GraphPad, La Jolla, CA, US). One-way ANOVA followed by Šidák multiple comparisons tests were performed (*). Comparison between only two independent groups was evaluated using an unpaired Student’s t*-test* (#). Differences in *p* values ≤0.05 were considered to be statistically significant.

## 3 Results

### 3.1 Optical characteristics and differentiation markers in 3D muscle cell model

**FIGURE 1:**
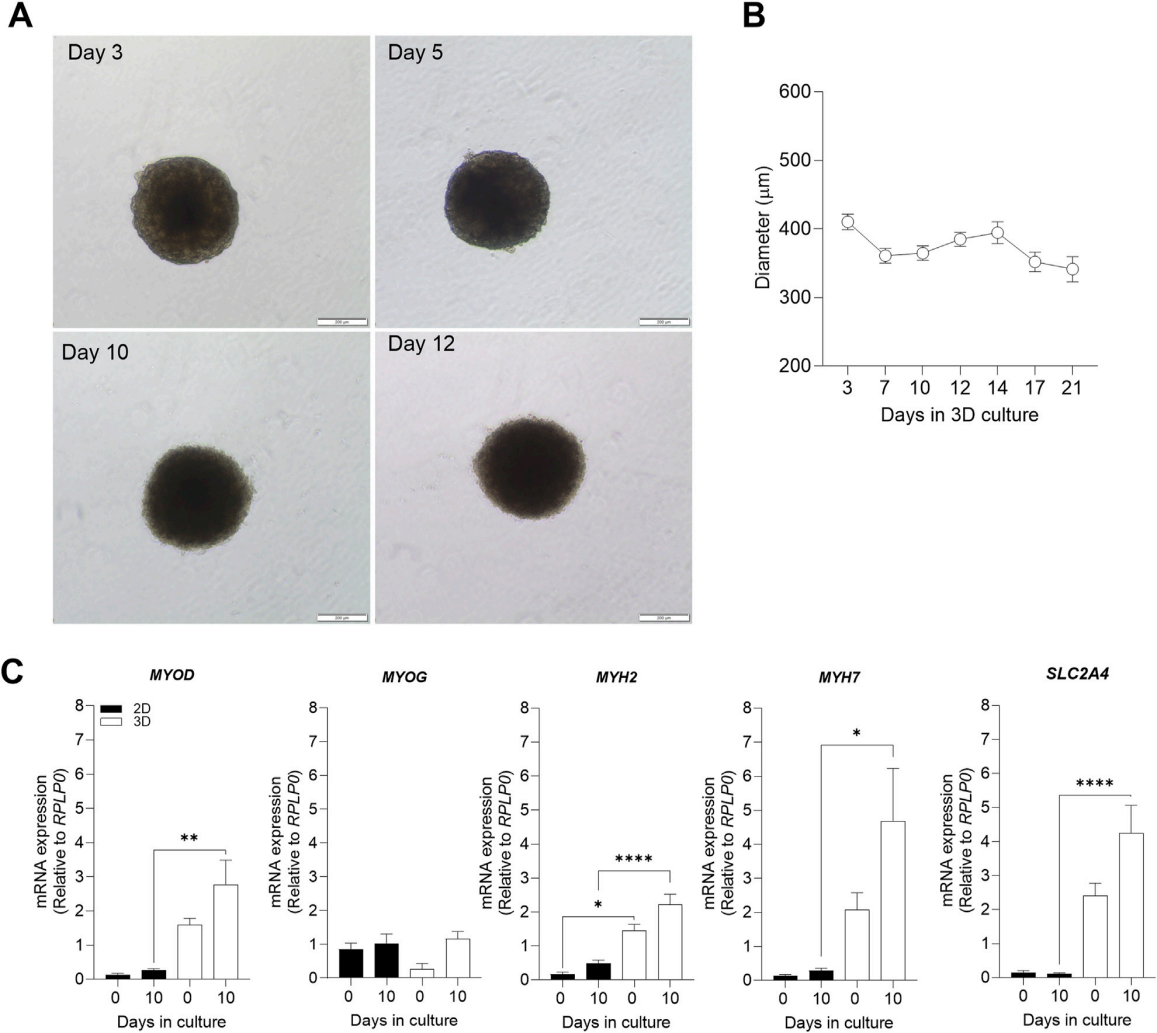
Evaluation of 3D morphological parameters and comparison of muscle cell differentiation markers in 2D and 3D models. Myospheres were formed in the ultra-low attachment treatment (ULA) 96-well plate system and differentiation was carried out between 0 and 21 days. After 10 days of differentiation, mRNA was isolated, and gene expression was analyzed by qPCR. **(A)** Phase-contrast photos of myospheres during 3, 5, 10 and 12 of differentiation. Diameter **(B)** was analyzed by AnaSP for up to 21 days of differentiation. **(C)** mRNA expression of the muscle differentiation markers *MYOD*, *MYOG*, *MYH2*, *MYH7*, and *SLC2A4* before (day 0) and after differentiation (day 10), normalized to housekeeping gene (*RPLP0*). Scale bar = 200 μm. Results are presented as mean ± SEM. *p < 0.05 **p < 0.01 ****p < 0.0001 by ordinary one-way ANOVA test.

To establish 3D cell culture model of human skeletal muscle cells (myoblasts), 3 × 10^4^ myoblasts were aggregated as spheroids and incubated in proliferation medium for 24 h. Spheroids with clear edges were formed within 24 h. Thereafter, the medium was replaced by differentiation medium and optical morphology characteristics and differentiation markers were monitored in the 3D skeletal muscle cell model. Changes in spheroid size were observed over time by analyzing the diameter of spheroids. During the first days of culturing (days 3–7), myospheres sizes reduced until it stabilized (from days 7–21) (Figures 1A, B). As a result, the subsequent experiments were performed at 10 days of differentiation, once the size of the spheroids was constant. Furthermore, due to biological differences between 2D and 3D cell models (Hess et al., 2010), we wanted to compare the two cell models and confirmed that changes in 3D myosphere model gene expression levels followed cell transition from myoblast to myotubes in comparison to the 2D myotubes model.

The process of muscle differentiation is regulated through different phases which stimulate myoblasts into fusion and maturation to become myotubes (Schmidt et al., 2019; Isesele and Mazurak, 2021). The differentiation process is initiated by an increased expression of myogenic differentiation 1 (*MYOD*) which induces gene expression of myogenin (*MYOG*) and subsequent expression of differentiation markers such as the myosin-heavy chains 2 (*MYH2*) and 7 (*MYH7*), and maturation factors related to metabolic muscle function like the insulin-regulated facilitative glucose transporter, solute carrier family 2 member 4 (*SLC2A4*). Comparison of expression of *MYOD*, *MYH2*, *MYH7*, and *SLC2A4* revealed higher mRNA expression levels in 3D than 2D myotube models (Figure 1C). This relevant finding demonstrated a higher efficiency of cell maturation during differentiation in 3D than in 2D myotube models. In both cell models, mRNA expression levels of *MYOD*, *MYOG*, *MYH2*, *MYH7*, and *SLC2A4* tended to increase after 10 days of differentiation (Figure 1C).

### 3.2 Cell viability and hypoxia core in 3D myosphere model

**FIGURE 2:**
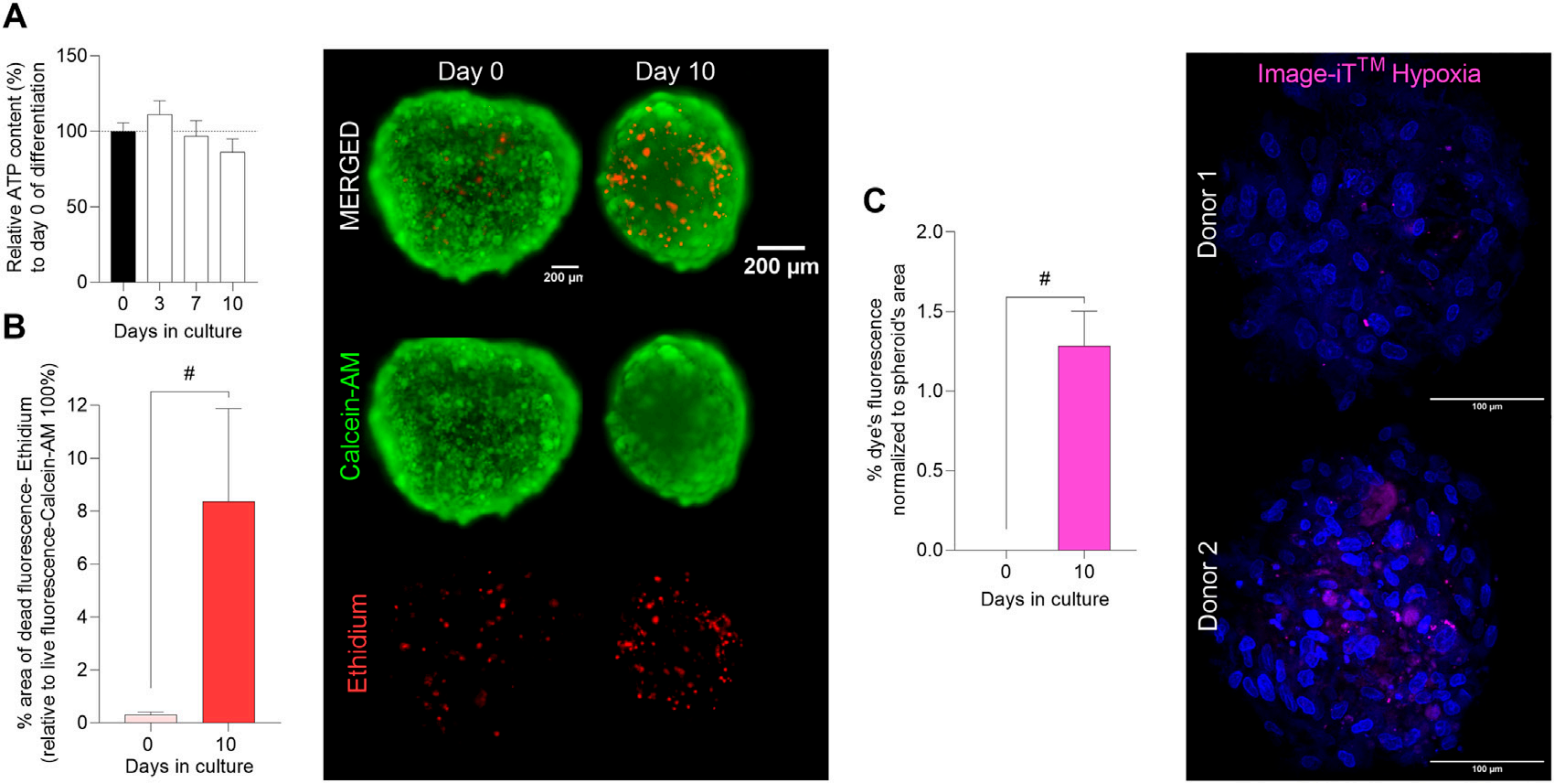
Evaluation of cell viability and hypoxia core in 3D muscle spheroids. Myospheres were formed in the ultra-low attachment treatment (ULA) 96-well plate system and differentiation was carried out between 0 and 10 days. **(A)** Myospheres viability was monitored as relative ATP content (%) (normalized to day 0) up to 10 days of differentiation by luminescence assay CellTiter-Glo 3D^®^ cell viability. **(B)** Area percentage (%) of dead (ethidium red) cells normalized to the area of live cells (calcein AM green) (ethidium area*100/calcein AM area) analyzed at days 0 and 10 of differentiation by the LIVE/DEAD^®^ Viability/Cytotoxicity Assay and representative images (scale bar = 200 µm). **(C)** % of hypoxia levels (Image-IT™ (magenta)) per spheroid’s area and representative images of the Image-IT™ dye staining (scale bar = 100 µm). Results are presented as mean ± SEM. #*p* < 0.05 by unpaired *t-test*.

To further characterize the 3D myosphere model, cell viability and hypoxia core were evaluated over differentiation time in the 3D skeletal muscle cell model. To analyze the viability of the spheroids after 10 days of differentiation, two different approaches were performed; relative ATP content and LIVE/DEAD staining. The relative ATP content assay did not show any significant differences in viability of the myospheres during the 10 days of differentiation (Figure 2A). LIVE/DEAD staining showed that cell viability inside the myospheres was reduced after 10 days. At day 0 of differentiation, a weak signal of dead cells was detected in the images accounting for around 0.5% of the area of live cells (defined by calcein AM fluorescence). However, after 10 days in culture, the analysis of dead cells (area) was increased up to 8% of the total area (Figure 2B).

Since cell death was mainly localized to the center of the myospheres (Figure 2B image), we analyzed the hypoxic levels during the differentiation phase. The results showed that fluorescence of Image-iT™ Green Hypoxia Reagent was not detectable at day 0 of differentiation, 24 h after spheroid formation (Figure 2C). After 10 days, the staining became detectable, and as expected, the dye localized to the middle of the myospheres (Figure 2C image). An average of 1.3% hypoxia was found by analysis of images from myospheres after 10 days in culture (Figure 2C).

### 3.3 Characterization of the internal 3D myosphere structure

**FIGURE 3:**
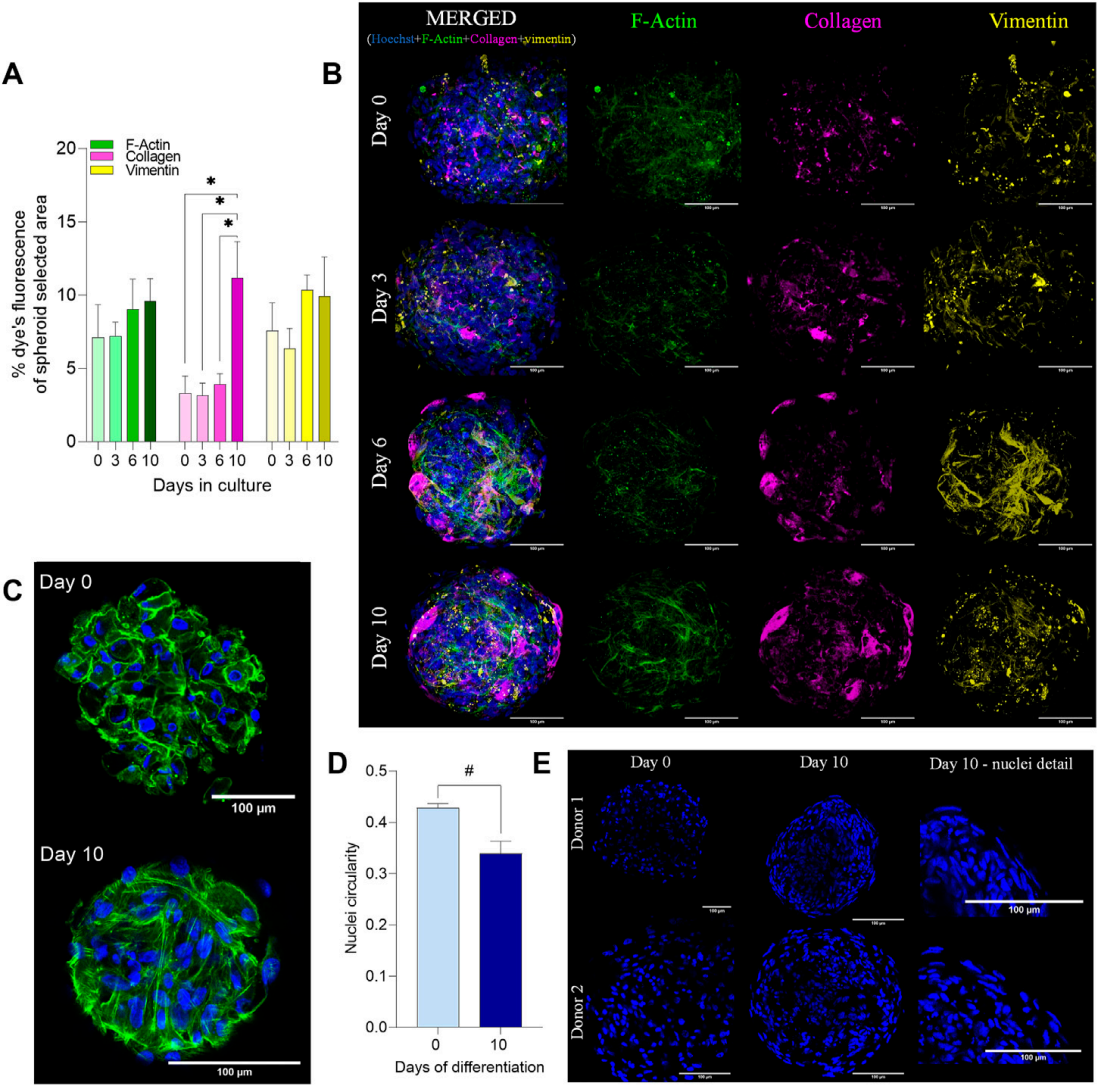
Characterization of the internal muscle spheroid structure. Myospheres were formed, differentiated up to 10 days, and fixed in 4% of paraformaldehyde (PFA) previous staining. **(A)** Analysis of structural markers represented the percentage (%) of dye in the total spheroid area over culture time. **(B)** Representative maximum intensity projection of the structural markers: F-actin (green; actin filaments), collagen (magenta; matrix production), and vimentin (yellow; vimentin filaments). **(C)** Representative image of F-actin before and after differentiation (days 0 and 10) (merge between nuclei (blue) and F-actin (green)). **(D)** Analysis of nuclei circularity (Hoechst) at 0 and 10 days of differentiation. **(E)** Representative fluorescence image sections from two different donors of nuclei staining at 0 (single rounded nuclei) and 10 (fused/elongated nuclei) days of differentiation. Scale bar = 100 µm. Results are presented as mean ± SEM. *p < 0.05 by ordinary one-way ANOVA test. #*p* < 0.05 by unpaired *t-test*.

In order to analyze the organization of the internal 3D skeletal muscle cell model structure over time (0–10 days) in culture, staining of nuclei and immunostaining of F-actin, collagen, and vimentin were performed. The amount of collagen showed a significant increase on day 10 compared to day 0, 3, and 6 of differentiation (Figures 3A, B). Collagen is produced inside the cells and secreted to the extracellular space as one of the main components of the ECM (Shen et al., 2021; Kruszewska et al., 2022). Through analysis of the images, it was possible to observe how collagen was produced homogeneously inside the myospheres during the first days. Nevertheless, on days 6–10, the staining was co-located within the external cells (spheroid’s superficial layer) (Figure 3B). Also for vimentin, localization in the spheroids changed during culturing. Vimentin is an intermediate filament from the cytoskeleton that takes part during myoblast development, i.e., in undifferentiated and proliferative cells, and disappears through myotube differentiation (Cizkova et al., 2009; El-Sheikh et al., 2022). A homogeneous punctured distribution of dye at days 0, 3 and 10 of differentiation followed by an external localization on day 6 (detected in the external layers) was observed (Figure 3B). Despite changes in localization, the results obtained from the analysis of overall vimentin staining showed no changes during the monitored time frame (Figure 3A).

Staining of F-actin showed no significant changes during the differentiation period (Figure 3A). However, the percentage of fluorescence area tended to increase during the last days of differentiation. F-actin is a cytoplasmic protein resulting from the assembled globular actin into a filament. Due to it being part of the primary component of the sarcomeric thin filament, it plays a necessary role in cell movement and muscle contraction among other functions such as intracellular transport and signaling (Henderson et al., 2017; Parker et al., 2020). The staining with F-actin as a structural/cytoplasmatic marker helped us to better understand the organization of the cells inside the sphere. During the first days of differentiation, the F-actin was mostly located around the cytoplasm (closer to the cell membrane), showing irregular circular shaped-cytoplasms. Throughout the differentiation, the staining became principally elongated and organized in multiple 3D directions, forming continuous filaments across several cells (cell fusion) (Figure 3C, Supplementary Figure S2 , Supplementary Video S1). Moreover, changes in nuclei morphology were observed during differentiation. At the beginning of differentiation, nuclei displayed a circular shape both inside and around the myosphere (known as isotropy). On day 10, we observed elongation of nuclei [evaluated by the decrease of nuclei circularity (Figure 3D)].

To demonstrate that the differences between the myospheres and the 2D muscle cell model are due to the structure and not because of an original poor cell culture obtained from donors, structural staining of the 2D cells was also performed (Supplementary Figure S3). As in the myospheres, actin, collagen and vimentin were stained in the cells in 2D format, however, any of them were significantly modulated through the differentiation (Supplementary Figure S3A). After 10 days of differentiation, both cell models underwent similar characteristics of muscle cell maturation, although the organization of the cytoskeleton was different between them. In the myospheres, F-actin was elongated but organized in multiple directions meanwhile in 2D model, F-actin was arranged in parallel from one cell to another (Figure 3C, Supplementary Figure S3B, S4).

Together with F-actin reorganization, the analysis of the nuclei morphology in the myospheres demonstrated a proportion of multinucleated cells with elongated and fused nuclei among the sphere (Figure 3E), providing evidence of muscle differentiation.

### 3.4 Functional characterization of 3D myospheres compared to 2D cell cultures

**FIGURE 4:**
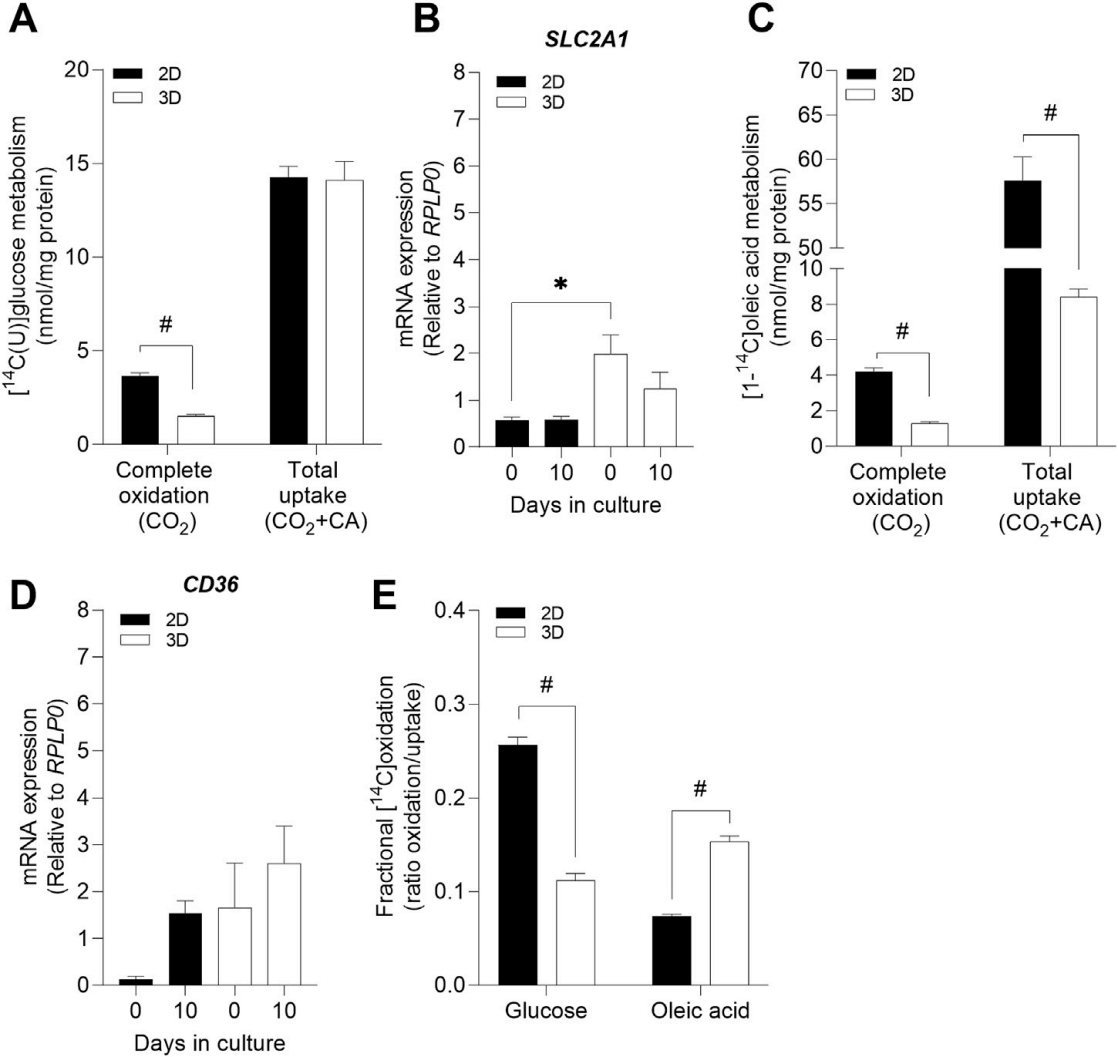
Comparison of glucose and oleic acid metabolism between 2D myotube cultures and 3D muscle spheroids. Myospheres were differentiated for 10 days and D-[^14^C (U]glucose (0.5 μCi/mL, 200 µM) or [1-^14^C]oleic acid (0.5 μCi/mL, 100 µM), respectively, were used in 4 h substrate oxidation assay to assess the metabolic profile. **(A)** Complete oxidation and total D-[^14^C (U]glucose uptake in 2D and 3D cell models. **(B)** mRNA expression of solute carrier family 2 member 1 (*SLC2A1*) (glucose transporter) at days 0 and 10 of differentiation, normalized to housekeeping gene (*RPLP0*). **(C)** [1-^14^C]oleic acid complete oxidation and uptake in 2D and 3D cell model’s metabolism. **(D)** mRNA expression of the fatty acid transporter *CD36* at days 0 and 10 of differentiation, normalized to housekeeping gene (*RPLP0*). **(E)** Fractional oxidation (ratio of substrate oxidation/uptake) of glucose and oleic acid in 2D and 3D cell models. Results are represented as mean ± SEM. #*p* < 0.05 by unpaired *t*-test; *p < 0.05 by ordinary one-way ANOVA test.

To study whether differences observed between the cell models could also have an impact on cellular energy metabolism, metabolic profile of the 3D myospheres and the 2D myotube cultures were assessed. Cellular uptake and oxidation were measured using labeled glucose and oleic acid as precursors. The results showed that myospheres oxidized less glucose than the 2D myotube model, even though the uptake of glucose was similar in both cell models (Figure 4A). Data obtained for glucose uptake were surprising due to the common gradient formed in the spherical shapes from the surface to the core (Valdoz et al., 2021). However, mRNA expression of solute carrier family 2 member 1 (*SLC2A1-*glucose transporter) was significantly higher in 3D compared to 2D at day 0, but not at day 10 of differentiation when the functional experiments were performed (Figure 4B). In contrast to glucose metabolism, myospheres showed lower oleic acid uptake and oxidation compared to 2D cultures (Figure 4C). Furthermore, despite lower oleic acid uptake in the myospheres, they showed similar mRNA expression of fatty acid transporter *CD36* as the 2D cells after 10 days of differentiation (Figure 4D).

Fractional substrate oxidation, which is the ratio between uptake and oxidation or how much of the substrate that is taken up that is in fact oxidized, was also determined. The reduced fractional glucose oxidation in myospheres indicated that the 3D muscle cell model was less glucose oxidative than the 2D model (Figure 4E). However, the same ratio calculated from oleic acid data showed that myospheres oxidized relatively more oleic acid than the 2D myotubes (Figure 4E. Thus, the myospheres incorporated/accumulated more glucose than they oxidize and oxidized a higher portion of the oleic acid taken up in comparison to the 2D myotube model.

## 4 Discussion

Although many differences between 2D and 3D cultures have been described, muscle tissue engineering has been mostly focused on exercise, muscle regeneration and drug discovery (Duval et al., 2017; Bremner et al., 2022; Jiang et al., 2022). The purpose of this work was to establish a simple method to create myospheres from human myoblasts as a 3D cell model to provide a platform to study skeletal muscle cell energy metabolism in healthy and diseased environments. The results of this work provide evidence of differences between 2D and 3D cell culture, suggesting that the format of muscle cell culture should be carefully considered when studying cell metabolism and working on disorders.

As previously described in literature, it is known that 3D cell models can be maintained *in vitro* for longer periods compared to 2D cell models (Jensen and Teng, 2020; Kang et al., 2021; Raffa et al., 2022). Besides, it has been reviewed that myospheres propagated *in vitro* as free-floating clusters can be extended over longer periods because of cell-cell interactions that maintain the sphere-like structures (Dessauge et al., 2021). In our routine laboratory procedures, myotubes for 2D experiments are kept below passage 5 and not differentiated for more than 7–8 days. However, on day 7 of differentiation, the myospheres have just reached a stable size (compact structure) and may not be sufficiently differentiated. As a consequence, we used the same cells for 2D and 3D experiments, and we differentiated the cells for 10 days since a particular purpose of this work was to compare 2D myotube and 3D myosphere models.

Myospheres were formed in less than 24 h by self-aggregation, and the cells were alive for at least 10 days of culturing. Differentiation and maturation markers were upregulated in the 3D model compared to 2D cells and characteristics of differentiated myotubes (multinucleated, elongated, orientated in multiple directions and fusion) were observed in the 3D structures (as shown in Figure 3). Moreover, the functional studies revealed different metabolic patterns between both cell models.

One of the first aspects to study when developing 3D culture is the size and the morphological structure. As cells are self-aggregated in spheroid culture, a gradient of nutrients is formed, reducing the availability of nutrients from the superficial layer to the inner core (Valdoz et al., 2021). The size of these spheroid-like tissues is important because when the diameter is between 500–1,000 μm, the lack of oxygen to the inner core (reduced diffusion) of these structures increases the levels of hypoxia and cause increased cell mortality (Costa et al., 2016; Marimuthu et al., 2018; Shen et al., 2021). Optical structure and size of the spheroids were monitored over time. The area and the diameter decreased over the first days, reaching a stabilized size from day 7 in culture. This phenomenon could be explained by the spheroid aggregation process. It has previously been described that spheroids undergo characteristic self-assembly after seeding (Shen et al., 2021). This process is divided into phases, from loose cells to a compact spheroid involving ECM production and cell-cell connection. Even though, this transition could be measured at molecular level by the amount of cell-cell connecting protein expression, the self-aggregation phases of spheroids were optically visible and measurable in terms of size.

ECM is important for spheroid assembly and maintenance since it constitutes the non-cellular physical support for the cells in tissues-like formats (Habanjar et al., 2021; Valdoz et al., 2021). It is established that ECM production also plays an important role in induction of myoblast differentiation (Jung et al., 2015). To further investigate the structure of the myospheres related to ECM and cell-cell connections, internal (immunofluorescence) structural markers were analyzed. Collagen is one of the principal components of ECM among others and it contributes to elastic, support, and strength properties of the body (Shen et al., 2021). The results of staining showed a significant increase in collagen expression after 10 days of differentiation. Interestingly, localization of collagen inside the myospheres varied through the differentiation phase, from homogenously distributed through the image sections to localization near the surface at day 10. Endogenous ECM production is a remarkable aspect of our myospheres since no external compounds were added to support the 3D structure and its differentiation (Marquette et al., 2007; Habanjar et al., 2021). Furthermore, increased collagen expression through differentiation may play a vital role in organizing the spheroid’s structure. In skeletal muscle, collagen type I is mainly secreted by fibroblasts which are primarily responsible for regulation of ECM and are usually found next to myogenic satellite cells (Kosnik et al., 2001; Gumucio et al., 2015; Ostrovidov et al., 2019; Habanjar et al., 2021). A previous study has described the relevance of using heterogenous multicellular spheroids to support crosstalk between cells and evaluate the impact of different cellular phenotypes on cell-cell interactions, ECM modulation and secreted factors (Lotsberg et al., 2022). In adult skeletal muscle, the intermediate filament vimentin has been positively used to identify fibroblasts in connective tissue (Cizkova et al., 2009; Tarbit et al., 2019). Since our 3D model is based on myoblasts originating from unsorted myoblast cell isolation (SVF), a certain proportion of non-muscle cell-like fibroblasts is expected in the myospheres. However, a specific culture medium for myoblasts proliferation was used during the isolation procedure that reduces the final proportion of fibroblast to around 15%–20% in our cell cultures (Supplementary Figure S1). Our results showed that vimentin was detected and consistently stained over time which means there was not an increase/proliferation of fibroblast in the myospheres. Certainly, it has been shown that fibroblasts have limited growth in scaffold-free cultures due to senescence (Valdoz et al., 2021). One of the main reasons to obtain non-purified cell cultures is because this procedure has gained importance as it provides a more physiological model (Murphy et al., 2011). Also, one of our collaborators has seen no major impact of different techniques to obtain and select myotubes *in vitro* in their myogenic capacity to differentiate into functional myotubes*.* In addition, several metabolic parameters were measured in either flow cytometry-immunoselected/sorted CD56^+^ cells or non-inmunoselected/separated cells, and no major effects of immunoselecting were found for glucose or fatty acid oxidation (Cedric Moro and Virginie Bourlier, Metabolic and Cardiovascular Research Institute, Toulouse, France, unpublished data).

**FIGURE 5:**
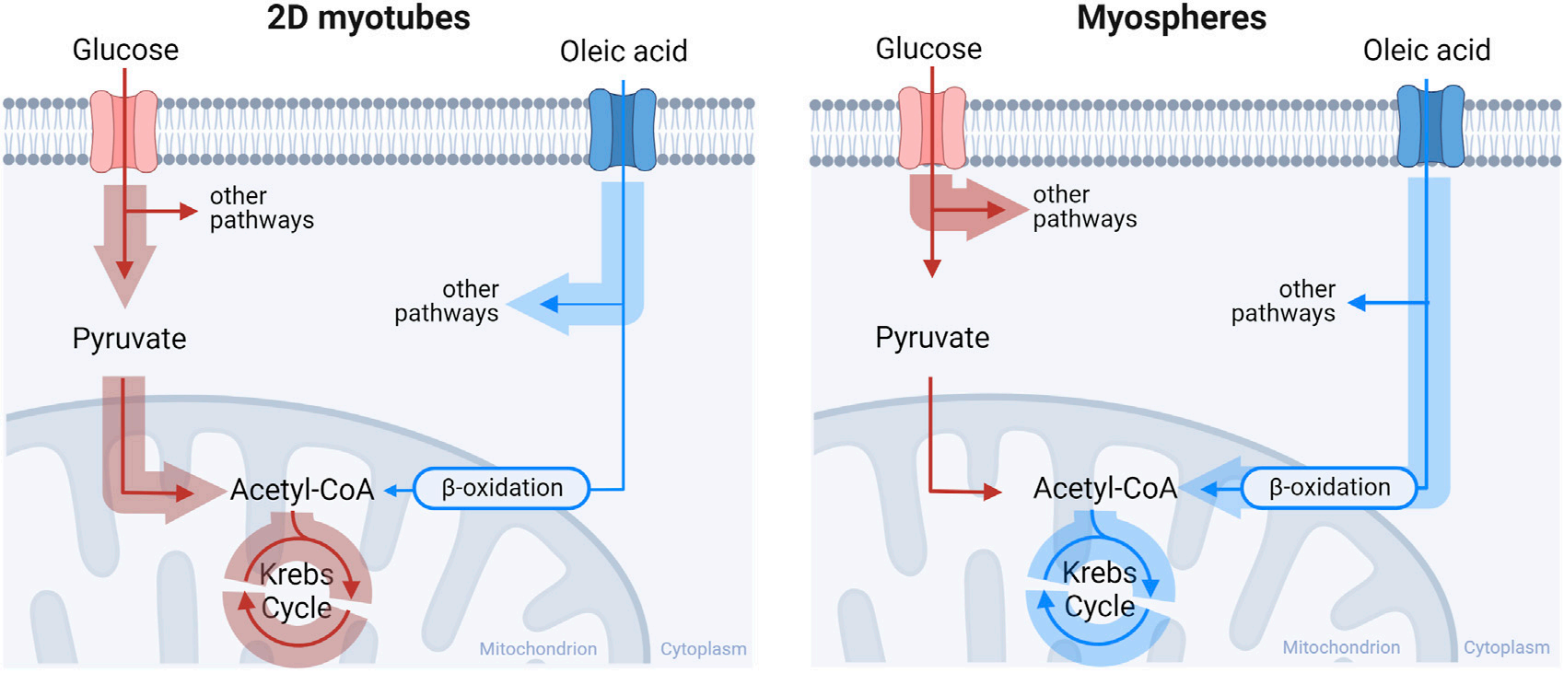
Pathway representation of glucose and oleic acid metabolism between 2D myotube and 3D myospheres culture. The 2D myotubes model had a preference to oxidize glucose while the 3D myospheres showed a preference towards lipid oxidative metabolism over glucose Made with Biorender.com.

The main structural aspects related to muscle differentiation are organized cytoskeleton, cell elongation and multinucleated myotubes formed by cell fusion processes (Chal and Pourquie, 2017). Staining of F-actin and nuclei helped us to further visualize these muscle characteristics in the internal structure of the myospheres. Cytoplasms and nuclei of the 3D model were elongated and fused, with cells showing multiple nuclei inside. In general, this anisotropic distortion of both structures is usually driven (stressed) by the cytoskeleton (F-actin) and can be used to detect the differentiation of myoblasts (Bray et al., 2010; Tremblay et al., 2013; Kung et al., 2018). Furthermore, the cytoskeleton of myospheres was also stretched and organized in multiple directions which is remarkable since the 3D muscle cell model stands for a spherical shape. In spite that myotubes should be aligned in parallel, these parameters revealed a comparable internal structure between differentiated myospheres and skeletal muscle. However, these characteristics are not only seen in 3D or *in vivo* models but are also easily observed in 2D cell models (Gaster et al., 2001b). To study the differentiation status of the myospheres, we examined some molecular markers associated with the muscle cell differentiation process. Gene expression of some myogenic differentiation and maturation markers was modulated by the type of cell culture. Thus, *MYOD, MYH2, MYH7* and *SCL26A4* mRNA expression levels were higher in the 3D myospheres compared to the 2D myotube model. Along the same line, it has previously been described that gene expression levels of *MYOD*, *MYOG* and MYHs were higher in 3D skeletal muscle cell models in comparison to 2D (Alave Reyes-Furrer et al., 2021). However, other authors have shown a similar mRNA expression of *MYOG* in differentiated 2D and 3D muscle cell models (Mudera et al., 2010). In our case, even though there were also no differences in *MYOG* mRNA expression between the compared models, the 3D structure clearly enhanced the rate of differentiation compared to the 2D platform based on the higher expression of *MYH2, MYH7* and *SCL26A4* differentiation markers. Furthermore, the cells grown in a 3D structure expressed higher amount of the slow fiber type genes (*MYH3*, *MYH8*, *MYH7*) compared to the fast fiber types (*MYH1* and *MYH2*) (Mudera et al., 2010; Alave Reyes-Furrer et al., 2021). Another characteristic of our 3D myosphere model compared to 2D myotube model, was the increased expression of *SCL26A4*. The expression of insulin-responsive glucose transporter (*SCL26A4*) is an indicative feature of mature skeletal muscle that increases with differentiation (Al-Khalili et al., 2003). It has previously been described that *in vitro* 2D cultured primary human myotubes have a low expression of *SCL26A4* compared with *in vivo* expression in adult skeletal muscle and, therefore, decreased glucose uptake stimulated by insulin availability (Al-Khalili et al., 2003; Aas et al., 2013). Our 3D myospheres had increased gene expression of *SCL26A4*, but further experiments need to be performed to confirm a possible enhanced insulin-induced glucose uptake in this cell model. The increased level of *SCL26A4* expression is in concordance with the increased expression of the slow fiber type marker (*MYH7*) which is also seen *in vivo* (Aas et al., 2013). Thus, the higher mRNA expression of differentiation and maturation markers in our 3D myospheres compared to the 2D myotubes is of importance since these results indicate that the 3D myospheres are more representative of adult skeletal muscles. In addition, glucose and oleic acid metabolism were different in the 3D myospheres *versus* the 2D myotube model. Considering our data, 2D cell muscle models seemed to be more glycolytic (using glucose as a source for mitochondria) while the cells grown in a 3D structure had a metabolic profile towards lipid oxidation (using oleic acid as a substrate for mitochondria) (Figure 5). This result also aligns with the previously described gene expression since fast fiber type muscles are known to be mostly glycolytic and slow fiber types are more oxidative (Al-Khalili et al., 2003; Aas et al., 2013). Although our results showed metabolic differences between myotubes cultured either in 2D cell model or as myospheres, some limitations of this study should be considered. We are aware that the limited representation of multinucleated cells could have limited suitability for mimicking the metabolic activity of physiological muscle. Also, the characterization of muscle contractability could provide valuable additional information about the 3D model. Since active contraction may help in the differentiation and maturation of the cells, an increased number of multinucleated cells could be expected as well. Thus, these limitations are a shortcoming of the study which requires further research.

Altogether, gene expression and metabolic data demonstrate an increased maturation phenotype of muscle cells in 3D culture that improves the relevance for *in vivo* representations and reproduce a more suitable tool for energy metabolism and disease modeling studies.

## Conclusion

We have demonstrated that the 3D human primary myosphere model presents an advantageous differentiation profile which may be consider for the study of metabolic disorders compared to the more commonly used 2D myotube cell cultures. A particular advantage of our 3D myosphere model is the ability to successfully generate multicellular and differentiated spheroids in a simple, low-cost, scaffold-free, and reproducible way in a very short time.

## Data Availability

The raw data supporting the conclusion of this article will be made available by the authors, without undue reservation.
