# Correction: Development of three-dimensional primary human myospheres as culture model of skeletal muscle cells for metabolic studies

**DOI:** 10.3389/fbioe.2025.1687822

**Published:** 2025-09-16

**Authors:** Andrea Dalmao-Fernandez, Aleksandra Aizenshtadt, Hege G. Bakke, Stefan Krauss, Arild C. Rustan, G. Hege Thoresen, Eili Tranheim Kase

**Affiliations:** ^1^ Section for Pharmacology and Pharmaceutical Biosciences, Department of Pharmacy, University of Oslo, Oslo, Norway; ^2^ Hybrid Technology Hub Centre of Excellence, Faculty of Medicine, University of Oslo, Oslo, Norway; ^3^ Department of Pharmacology, Institute of Clinical Medicine, University of Oslo, Oslo, Norway

**Keywords:** skeletal muscle, myosphere, energy metabolism, metabolic disorders, 3D cell model, muscle spheroid

There was a mistake in Figure 1 as published. One of the gene names is misspelled: “solute carrier family 26 member 4 (*SLC26A4*)”, the correct form is: “solute carrier family 2 member 4 (*SLC2A4*).” The corrected Figure 1 appears below.

**FIGURE 1:**
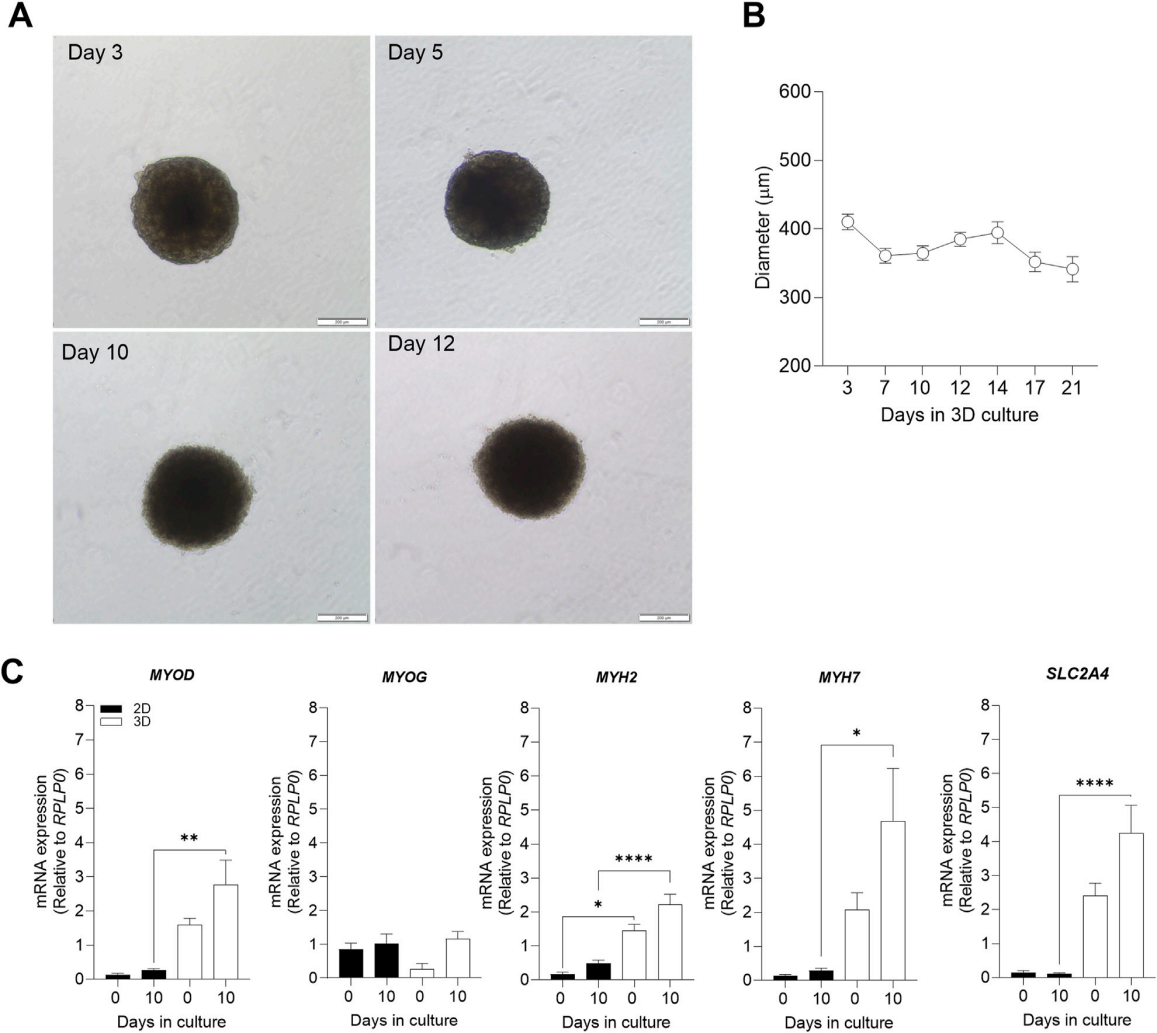
Evaluation of 3D morphological parameters and comparison of muscle cell differentiation markers in 2D and 3D models. Myospheres were formed in the ultra-low attachment treatment (ULA) 96-well plate system and differentiation was carried out between 0 and 21 days. After 10 days of differentiation, mRNA was isolated, and gene expression was analyzed by qPCR. **(A)** Phase-contrast photos of myospheres during 3, 5, 10 and 12 of differentiation. Diameter **(B)** was analyzed by AnaSP for up to 21 days of differentiation. **(C)** mRNA expression of the muscle differentiation markers *MYOD, MYOG, MYH2, MYH7*, and *SLC2A4* before (day 0) and after differentiation (day 10), normalized to housekeeping gene (RPLP0). Scale bar = 200 μm. Results are presented as mean ± SEM. *p < 0.05 **p > 0.01 ***p < 0.0001 by ordinary one-way ANOVA test.

There was a mistake in the caption of Figure 1 as published. One of the gene names is misspelled: “solute carrier family 26 member 4 (*SLC26A4*”), the correct form is: “solute carrier family 2 member 4 (*SLC2A4*)”. Also the panels aren’t correct: panel D should not be there. The corrected caption of Figure 1 appears below.

In the first section of the **Results** (*3.1 Optical characteristics and differentiation markers in 3D muscle cell model*), one of the gene names is misspelled: “solute carrier family 26 member 4 (*SLC26A4*)”, the correct form is: “solute carrier family 2 member 4 (*SLC2A4*)”.

A correction has been made to the section **Results**, *subsection 3.1 Optical characteristics and differentiation markers in 3D muscle cell model*, Paragraph 2:

“The process of muscle differentiation is regulated through different phases which stimulate myoblasts into fusion and maturation to become myotubes (Schmidt et al., 2019; Isesele and Mazurak, 2021). The differentiation process is initiated by an increased expression of myogenic differentiation 1 (*MYOD*) which induces gene expression of myogenin (*MYOG)* and subsequent expression of differentiation markers such as the myosin-heavy chains 2 (*MYH2*) and 7 (*MYH7*), and maturation factors related to metabolic muscle function like the insulin-regulated facilitative glucose transporter, solute carrier family 2 member 4 (SLC2A4). Comparison of expression of *MYOD,* *MYH2,* *MYH7,* and *SLC2A4* revealed higher mRNA expression levels in 3D than 2D myotube models (Figure 1C). This relevant finding demonstrated a higher efficiency of cell maturation during differentiation in 3D than in 2D myotube models. In both cell models, mRNA expression levels of *MYOD, MYOG, MYH2, MYH7*, and *SLC2A4* tended to increase after 10 days of differentiation (Figure 1C)”.

The original article has been updated.

