# Supplementary material for: Development of three-dimensional primary human myospheres as culture model of skeletal muscle cells for metabolic studies
Source: Front Bioeng Biotechnol. 2023 Mar 23;11:1130693. doi: 10.3389/fbioe.2023.1130693 (PMC10076718; doi:10.3389/fbioe.2023.1130693)
Supplement: Supplementary file 3 [file DataSheet1.PDF]

Supplementary Material

Development of three-dimensional primary human myospheres as culture model of skeletal muscle cells for metabolic studies.

Andrea Dalmao-Fernandez[1*], Aleksandra Aizenshtadt[2], Hege G. Bakke[1], Stefan Krauss[2], Arild C. Rustan[1], G. Hege Thoresen[1,3], Eili Tranheim Kase[1]
***Correspondence:**

Andrea Dalmao-Fernandez

A.d.fernandez@farmasi.uio.no

1 Supplementary Data

1.1 Supplementary Figure

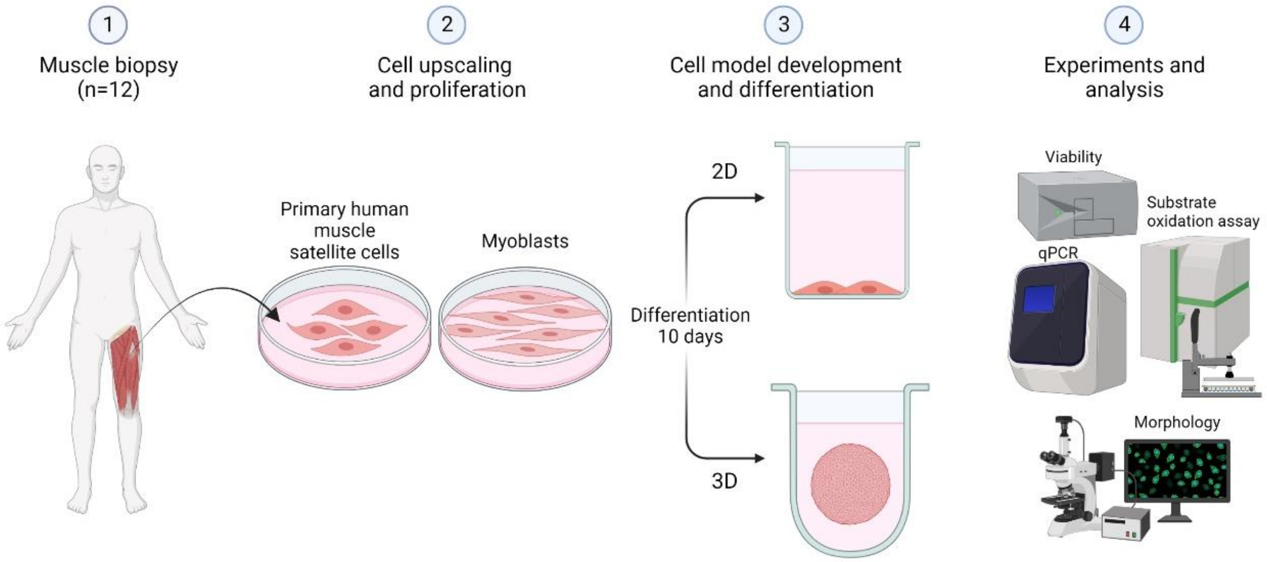

Supplementary Figure 1. Workflow

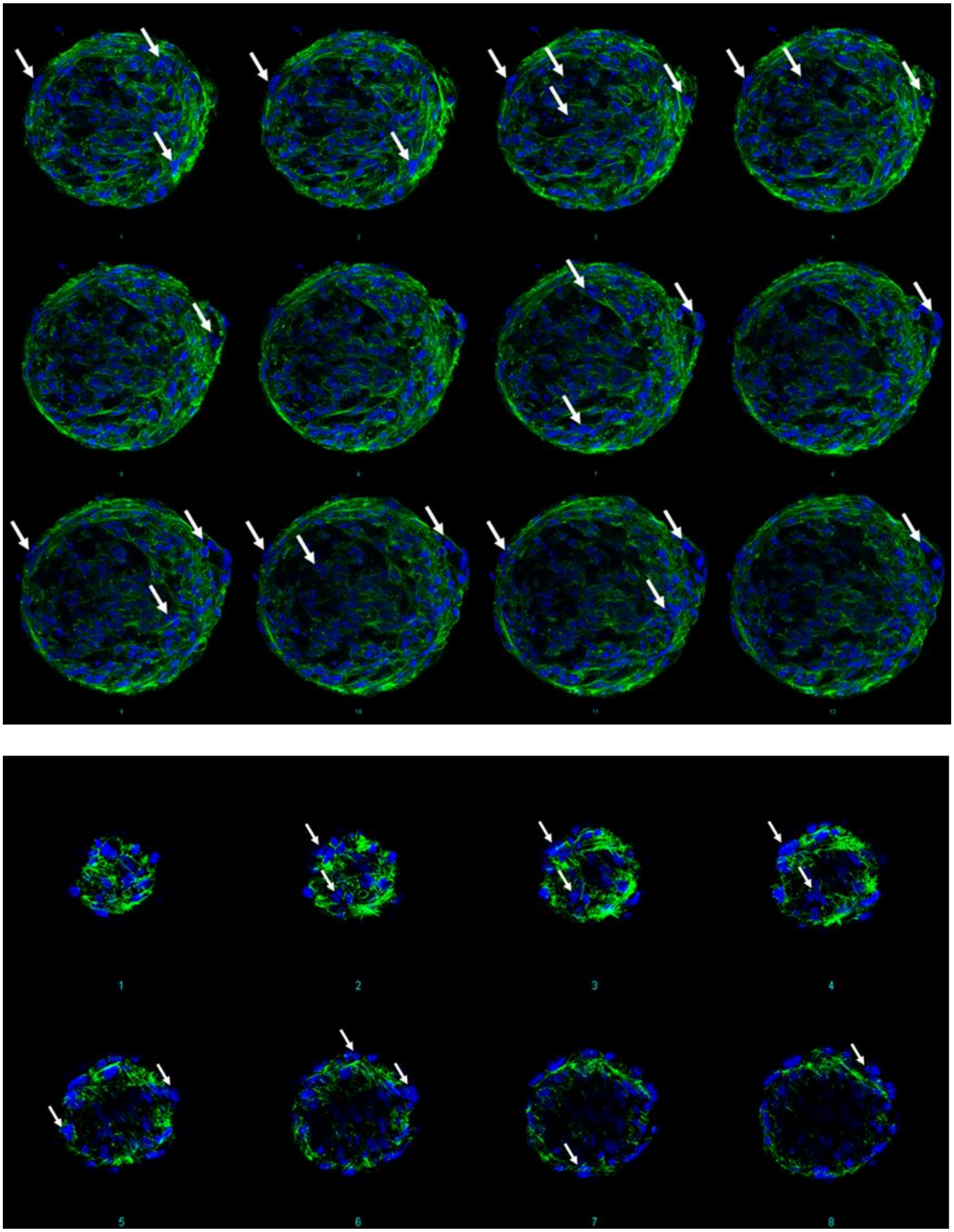

Supplementary Figure 2. Sequential analysis of multinucleated cells in Z-stacks of 2 different myospheres. Myospheres were formed, differentiated up to 10 days, and fixed in 4% of paraformaldehyde (PFA) previous staining. Representative Z-stacks merge of the structural markers: F-actin (green; actin filaments) and nuclei (blue). White arrows point to multinucleated cells (more than 2 nuclei within cytoskeleton (actin)).

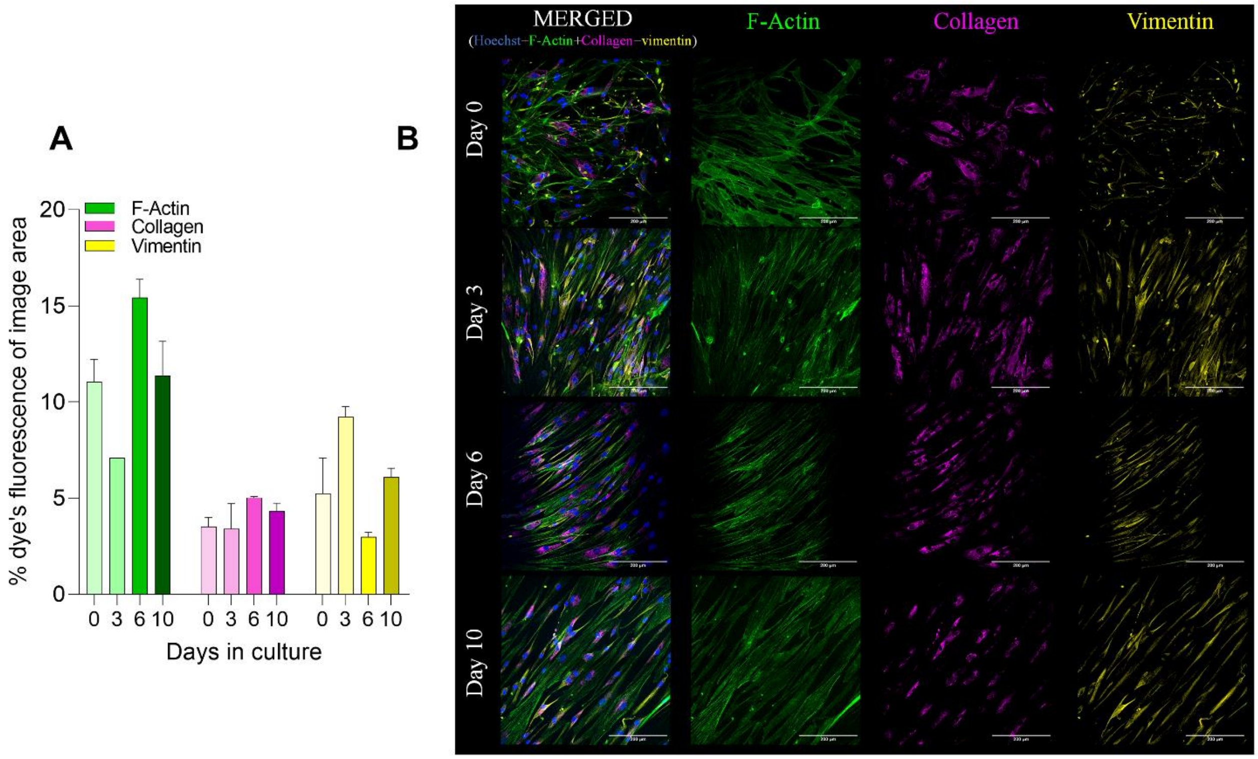

Supplementary Figure 3 Characterization of cell structure in 2D muscle cell model. Myoblasts were cultured and differentiated to myotubes up to 10 days, and fixed in 4% of paraformaldehyde (PFA) previous staining. The staining was performed following the same protocol and in the same donors as the myospheres. (A) Analysis of structural markers represented the percentage (%) of dye in the total photo area over culture time. (B) Representative maximum intensity projection of the structural markers: F-actin (green; actin filaments), collagen (magenta), and vimentin (yellow; vimentin filaments). Scale bar = 100 µm. Results are presented as mean ± SEM.

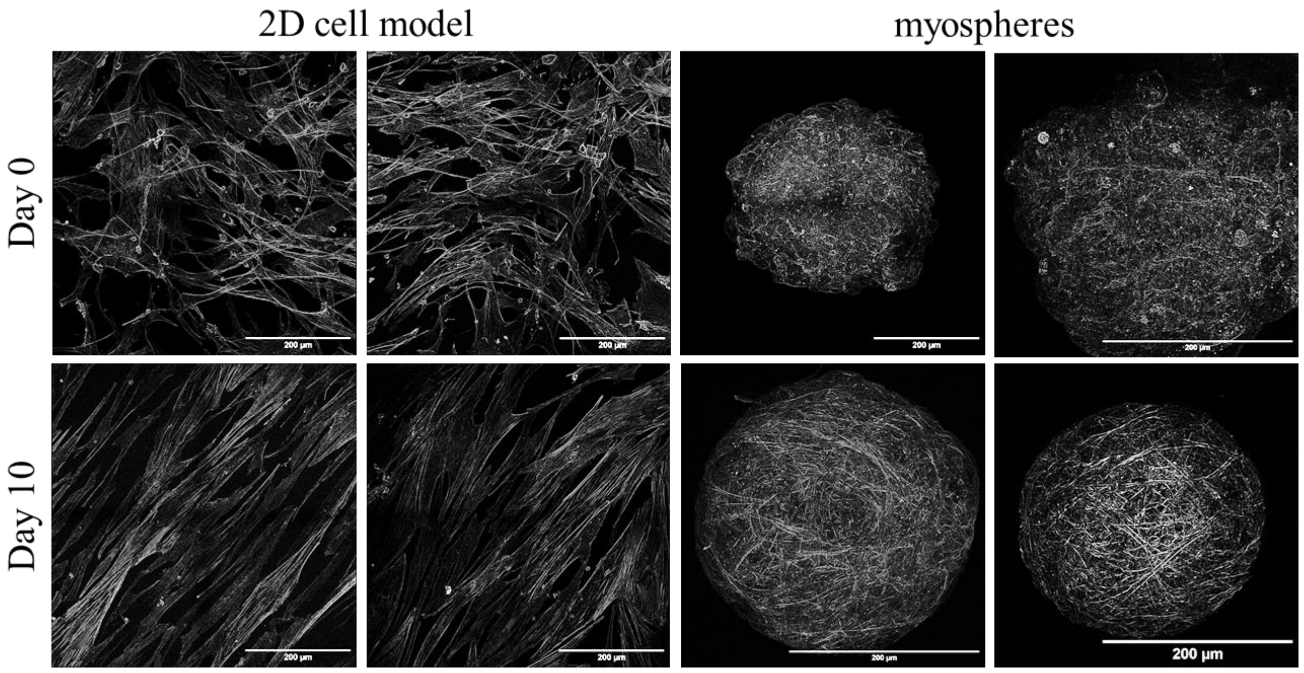

Supplementary Figure 4. Representative images of F-actin filaments organization in two different donors of 2D cell model and myospheres. Images were taken before (day 0) and after (day 10) differentiation and analyzed by ImageJ, tubeness plugin. Scale bar = 200µm.

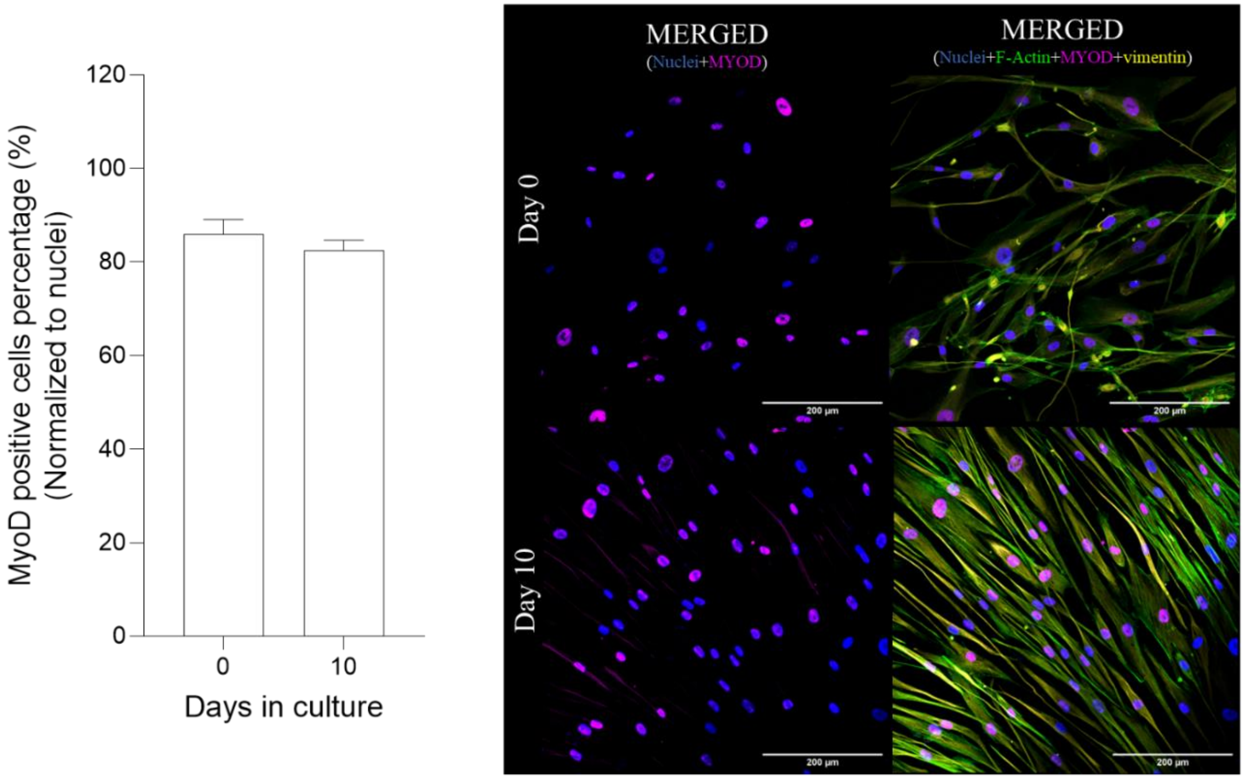

Supplementary Figure 5. Percentage of muscle cells in 2D cell culture. Muscle cells were cultured in a 2D platform and differentiated for 10 days. Before (day 0) and after (day 10) differentiation, nuclei (Hoechst, blue), cytoskeleton (F-actin, green), vimentin (yellow) and MYOD transcription factor were stained and images were analyzed with ImageJ. % of $MYOD^{+}$/$Hoechst^{+}$ was calculated compared to $Hoechst^{+}$ cells. Scale bar = 200 µm.
